# Ocular images-based artificial intelligence on systemic diseases

**DOI:** 10.1186/s12938-023-01110-1

**Published:** 2023-05-19

**Authors:** Yuhe Tan, Xufang Sun

**Affiliations:** Department of Ophthalmology, Tongji Hospital, Tongji Medical College, Huazhong University of Science and Technology, Wuhan, People’s Republic of China

**Keywords:** Artificial intelligence, Deep learning, Ocular images, Fundus photographs, Systemic diseases

## Abstract

**Purpose:**

To provide a summary of the research advances on ocular images-based artificial intelligence on systemic diseases.

**Methods:**

Narrative literature review.

**Results:**

Ocular images-based artificial intelligence has been used in a variety of systemic diseases, including endocrine, cardiovascular, neurological, renal, autoimmune, and hematological diseases, and many others. However, the studies are still at an early stage. The majority of studies have used AI only for diseases diagnosis, and the specific mechanisms linking systemic diseases to ocular images are still unclear. In addition, there are many limitations to the research, such as the number of images, the interpretability of artificial intelligence, rare diseases, and ethical and legal issues.

**Conclusion:**

While ocular images-based artificial intelligence is widely used, the relationship between the eye and the whole body should be more clearly elucidated.

**Supplementary Information:**

The online version contains supplementary material available at 10.1186/s12938-023-01110-1.

## Background

Artificial intelligence (AI) is a subfield of computer science in which computer algorithms are trained to perform tasks associated with human intelligence [1]. AI research encompasses a wide range of topics, including machine learning, deep learning, natural language processing, decision support systems, robotics, and many others. AI can learn from previous experiences, make sound decisions, and respond quickly. Because of this trait, AI is frequently employed in recommendation algorithms, search engines, autonomous driving, health care, and other industries, and has made significant progress.

The eye has unique anatomical structure—its transparent refractive interstitium, that permits light to pass through the pupil to the retina, allowing the eye to serve as a window to examine the state of the blood vessels and nerves. Systemic diseases, such as hypertension and diabetes, can manifest as distinct ocular presentations. The optical transparency of ocular structures enables non-invasive observation of changes in vasculature and nerves, a unique diagnostic capacity not available through alternative examination modalities. Therefore, the reflection of whole-body status based on ocular features has always been hot. With the advancement of AI techniques, images are commonly used in studies to diagnose ocular diseases such as diabetic retinopathy [2], age-related macular degeneration [3], retinopathy of prematurity [4], glaucoma [5], and others. Furthermore, the use of ocular images has been expanded beyond the study of ocular disorders and has aided in the discovery of various previously unknown connections with systemic diseases. AI can uncover a wealth of information that doctors previously couldn't see with their naked eyes, widening the breadth of disease diagnosis.

In recent years, there are many studies connecting ocular features with systemic diseases and risks, such as diabetes, cardiovascular disease, Alzheimer's disease, kidney disease, and so on. However, advances about ocular images-based AI on systemic diseases have yet to be summarized in detail. This review aims to highlight the recent progress in various systemic diseases.

## AI, machine learning, and deep learning

**Fig. 1:**
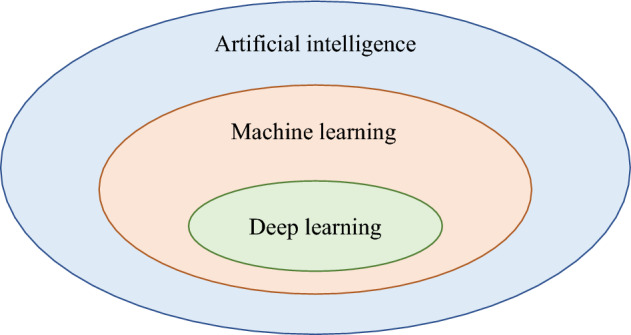
The relationship between artificial intelligence, machine learning and deep learning

AI seeks to make machines with human-like intelligence. Machine learning, as a subfield of AI, is often confused with the concept of AI. Machine learning refers to the extraction of features from data through a series of mathematical algorithms, leading to tasks such as prediction and classification [6]. Deep learning, a type of machine learning, is one of the most often utilized image-based technologies in the field of medical AI. Using a multi-layer structure, deep learning extracts more advanced features from the raw data. Each layer contains data processing units, often called neurons, that enable it to process large amounts of data simultaneously and obtain more abstract information, just as humans do [6]. Convolutional neural network (CNN), is one of the most common algorithms for deep learning. Convolutional layers are introduced to maintain the shape of the input data, which is especially important when the input are images. The pooling layers are used to compress the data while ensuring the invariance of the features. Figure 1 provides a brief statement of the relationship between AI, machine learning, and deep learning.

Machine learning algorithms can be broadly classified as unsupervised and supervised based on the different experiences in the learning process. The difference between them is whether the training samples are labeled or not. Moreover, depending on the continuity of the target data, the algorithms can be classified as classifiers and regressors. Classifiers are often seen in the diagnosis and classification of diseases, and regressors can be used for the prediction of numerical laboratory indicators. AI’s primary function in the medical area is to assist physicians in diagnosis, monitoring, and decision-making based on medical data. The three main categories of AI tasks are classification, clustering, and prediction [6]. Classification refers to the division of the input data into known groups. It is often used in the diagnosis of diseases. Clustering is also a grouping task, but unlike classification, clustering divides groups that were previously unknown. It can be applied to the grading of diseases. Prediction is the prediction of future parameters, such as predicting future risk of morbidity, or prognosis, based on historical data. Prediction algorithms require the use of longitudinal data, and there are fewer prediction studies due to the difficulty of obtaining data.

## Ocular images-based AI on systemic diseases

**Fig. 2:**
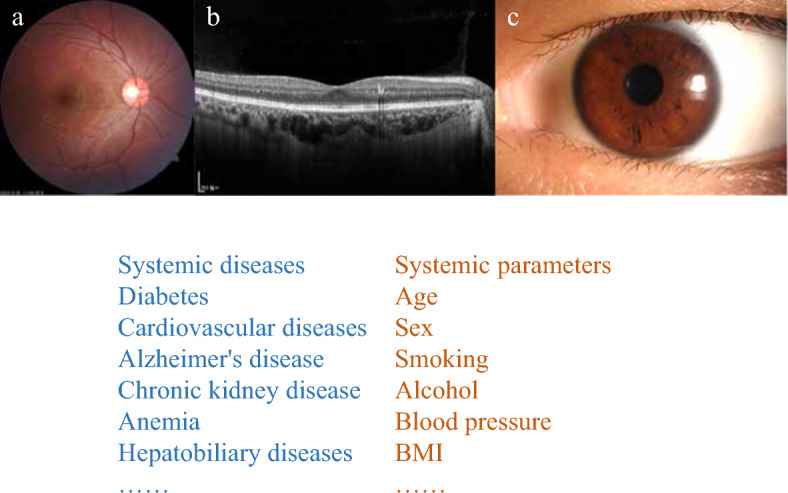
Overview of ocular images-based AI for systemic diseases and parameters. **a**–**c** Are three main kinds of ocular images used in artificial intelligence. **a** A retinal fundus photograph; **b** A optical coherence tomography image; **c** A slit-lamp image

Ophthalmology is an image-dependent field that generates a large number of images. Many imaging modalities, including retinal fundus photography, optical coherence tomography (OCT), OCT-Angiography (OCT-A), fluorescein fundus angiography, and anterior segment photography, are clinically available in ophthalmology. Since different diseases have diverse ocular manifestations, the researchers develop AI models using various ophthalmic images. AI research based on retinal fundus photographs are the most common due to their ease of access and large amount of data. The second is based on OCT, followed by anterior segment photographs. In AI studies of ocular images and systemic diseases, the majority of them are for the detection of systemic diseases using ocular images, with prediction accounting for a small percentage and clustering being rarely reported. Current studies focus not only on systemic diseases but also on biological parameters and risk factors (Fig. 2). The AI studies discussed in the review are summarized as Additional file 1: Table S1.

## Endocrine diseases

### Diabetes

The rising number of people with diabetes places a significant burden on global health. As a disease characterized by chronic hyperglycemia, diabetes can lead to many complications, such as retinopathy and nephropathy, and is associated with cardiovascular disease. Early screening for diabetes is a focus of health programs in many countries. Oral glucose tolerance test is the major tools for diagnosing diabetes. As the gold standard, its accuracy is high, but the invasive and frequent puncture blood collection operations limit its use in widespread screening. Noninvasive and convenient screening methods are needed.

There is no doubt about the relationship between diabetes and the eyes. Long-term chronic hyperglycemic environment brings oxidative stress, metabolic problems, inflammatory response, and many other disorders to the eye and causes various eye illnesses such as diabetic retinopathy, glaucoma, and cataract [7]. Diabetes patients' retinal microvasculature has already been changed compared to healthy individuals before they acquired diabetic retinopathy [8]. Thus, the detection of diabetes based on retinal images seems to be well documented. In 2020, retinal fundus photographs were collected and used to construct an AI system to detect hyperglycemia [9]. The accuracy of hyperglycemia detection was 78.7%, while the area under the receiver operating characteristic curve (AUC) was 0.880. However, the researchers selected only 1222 images of Chinese rural residents, and the small number of images may have a significant impact on the predictive efficiency of the model. In 2021, Zhang et al. used an AI system to predict diabetes, and over 100,000 images were used in the construction of the model [10]. A person could be screened for type 2 diabetes using fundus photographs. In addition, combining fundus photographs and patient metadata, they predicted the future incidence of diabetes in patients from the cohort study within 5 years. The use of longitudinal data to predict the future of healthy patients is what makes the work of Zhang et al. unique. In their work, rather than directly predicting the incidence of developing type 2 diabetes in healthy subjects within 5 years, the subjects were divided into three groups of low, medium and high risk by risk score. Zhang et al. used metadata and fundus images to predict the risk group of the test set. In this way, doctors can understand and inform patients of their future risk of developing the disease and take actions such as lifestyle changes to reduce the incidence. However, despite the cleverness of this method, it still has some shortcomings. The risk score used by Zhang et al. is not an internationally accepted standard for predicting the risk of developing diabetes. Therefore, the generalizability of this grouping is to be questioned. In external datasets, the AUC of detecting diabetes were over 0.80, and the AUC of prediction model was 0.824. Also, to increase the scope of application of the AI system, they loaded the system on a smartphone to screen the uploaded retinal images through the cloud service. AI based on mobile devices and cloud can broaden the scope of fundus images for screening, reducing the burden of care and regional disparities in healthcare resources.

## Cardiovascular diseases

Cardiovascular diseases are the leading cause of death worldwide [11]. The importance of early identification of cardiovascular diseases for patient health cannot be overstated. Hypertensive retinopathy is frequently employed as a biomarker representing hypertension in the field of ophthalmology. Furthermore, because the fundus vessels are unique in that they can be seen directly through the eye, numerous fundus vessel parameters such as vessel diameter, density, and degree of tortuosity were included in the research of cardiovascular diseases [12, 13]. With the help of AI, ocular and cardiovascular diseases research have become more in-depth.

Major cardiovascular risk factors, such as age, gender, blood pressure, smoking status, and so on were detected based on retinal images [14–16]. AI based on retinal images could get the risk factors directly, compared to repeated measurements and questionnaires. However, AI seemed to be overused in predicting the risk factors. The accuracy of the information from AI was significantly weakened. And it is not difficult to ask verbally or to measure the parameters. It is detrimental to the promotion of screening by AI based on retinal images. On the other hand, as a typical marker of cardiovascular events, coronary artery calcification fraction has also been detected by AI from fundus images. Son et al. grouped subjects according to coronary artery calcification fraction and age interval and compared the predictive efficacy of fundus images and clinical data [17]. The AUCs were 0.823, 0.832, and 0.828 with unilateral images, bilateral images, and age. According to the heatmap, the AI model focuses on the blood vessels of the retina. In the diagnosis of cardiovascular diseases by AI based on ocular images, hypertension and atherosclerosis are the major ones. Kim et al. developed an AI system with an AUC of up to 0.961 for hypertension [16]. The ability to access the risk of cardiovascular events is important for clinical work. Researchers want to achieve this based on the vascular status reflected by the eyes. Apart from detecting risk factor and diseases, AI based on ocular images was used to predict the incidence of cardiovascular events as well. Retinal images and clinical data were used to construct the prediction models. In these researches, atherosclerosis score and coronary artery calcification fraction detected by AI were used as predictors in the longitudinal studies [17–19]. Similar to the prediction of diabetes above, the investigators did not directly predict the incidence of cardiovascular events in the subjects, but instead used fundus images to predict atherosclerosis scores or coronary artery calcification scores. These two parameters were used to group subjects and to predict cardiovascular events in different groups. These studies reflect the possibility to obtain relevant biomarkers of cardiovascular disease from the fundus and to further predict the future progression of the disease with the help of these parameters.

## Neurological diseases

The eye is referred to as the “window to the soul” in literature, and the retina is an anatomical extension of the central nervous system. In reaction to internal and external environmental stimuli, the eye has a physiological and pathological state that is similar to that of the brain and spinal cord. As a result, research into the link between the eye and the neurological system is becoming increasingly common. For example, patients with Parkinson’s disease can also exhibit visual impairment. Their retinal ganglion cells (RGCs) are injured, and the retinal nerve fibers layer (RNFL) in the retina is thinning [20]. Similarly, alterations in the structure and function of the retina have been seen in people with Alzheimer's disease (AD). Aβ and pTau proteins were found in the retina of Alzheimer's disease patients, which were representative molecules of Alzheimer's disease pathogenesis. OCT has produced evidence for considerable thinning of the peripapillary NFL, macular volume loss, and nerve fiber density reduction in individuals with moderate to severe AD, indicating that thinning could develop early in disease progression [21]. Besides, among older adults, thinner RNFL thickness at baseline was associated with a greater decline in cognitive function scores at follow-up [22]. Given the retina's tight association with neurological illnesses, it's unsurprising that researchers have employed AI to investigate the eye's relationship with the neurological system.

### Alzheimer's disease (AD)

The diagnosis of AD relies on clinical manifestations, imaging, and cognitive-psychological examinations, which may occur in the late stages of AD. As the aging process accelerates, early screening for AD is necessary. Cheung et al. retrospectively collected fundus images from AD patients and healthy individuals in 11 studies and different countries, to construct and validate a model for the diagnosis of AD [23]. The AUCs of the external validation sets were from 0.73 to 0.91. They also found that the AI model was able to distinguish between beta amyloid-negative and positive patients, and had better performance in patients with ocular disease. Since retinal thickness correlates with AD [24], OCT images that present retinal thickness are a good source for constructing AI. The AI algorithm for texture acquisition from OCT images is well implemented for AD detection, with an AUC of 0.795 [25]. In addition, the combination of multimodal imaging and clinical data may improve the efficacy of AI systems. Based on this, multimodal retinal images including OCT, OCT-A, Ultra-widefield scanning laser ophthalmoscopy, and the patients’ data were combined to develop AI models to detect AD [26]. Ganglion cell-inner plexiform layer thickness map in OCT and combined model made good results of AUC over 0.8.

### Others

For a subset of neurological diseases that are difficult and costly to diagnose, AI based on retinal images has provided a convenient tool of screening. An AI system to diagnose diabetic peripheral neuropathy based on retinal color images was developed [27], which could provide a chance to screen their peripheral neuropathy status when people with diabetes screened their eyes. In patients with diabetic retinopathy, the AUC reached over 0.85. Also, the retinal vascular trajectory was acquired with the help of an AI algorithm, which connected the retina with schizophrenia and bipolar disorder, and performed the accuracy of 0.86 and 0.73, respectively [28]. Moreover, Lau et al. connected retinal images with MRI, and they detected white matter hyperintensities in healthy people, which was vital for the development of cerebral small vessel disease, and both of the sensitivity and specificity were over 0.9 [29].

## Kidney diseases

### Chronic kidney disease (CKD)

The kidney and the eye share similarities in structure, development, physiology, and pathogenic pathways [30]. Ocular manifestations, such as retinal microvascular parameters, have been shown to predict the development of CKD [31]. Kidney disease can also be associated with ocular abnormalities, such as tubulointerstitial nephritis uveitis syndrome (TINUS) [32, 33]. In children, TINUS presents with fever, pain, photophobia, and bilateral acute onset of non-granulomatous anterior uveitis associated with the diagnosis of interstitial nephritis, tubulointerstitial nephritis that often precedes or coincides with ocular symptoms. A study on the association of age-related macular degeneration (AMD) with CKD found that early AMD without geographic atrophy and choroidal neovascularization was three times more common in patients with moderate CKD than in those with mild or no CKD [34]. The diagnosis of kidney disease currently relies on kidney function, urine routine, and kidney puncture biopsy. These methods are slightly cumbersome, and we need some more concise screening methods.

A deep learning model was developed to predict early renal functional impairment based on retinal images, and researchers found that the model had a greater performance in patients with higher HbA1c levels (AUC over 0.81) [35]. Furthermore, the combined model of retinal images and clinical data detecting CKD showed better performance than the images-only model [36]. In the whole population, the AUC of combined model was about 0.8, while in patients with diabetes or hypertension, the AUC exceeded 0.9. AI could not only distinguish between healthy people and patients with CKD, and could also grade the CKD based on eGFR. In addition, AI based on retinal images in cohort studies, researchers predicted the progress of CKD in the future [37]. They used metadata, fundus images, and a combination of metadata and fundus images to construct predictive models and measure the risk of developing CKD and advanced CKD in healthy subjects. The prediction was also performed in groups with different risk stratification. Cox proportional hazards mode was applied, and the C-index of the combined model was 0.719. Prediction accuracy of up to 0.844 on the internal validation set. Of course, this research has limitations. To improve screening performance, researchers chose AI model with high sensitivity but low specificity, which could result in a large number of CKD misdiagnoses. The researchers' risk stratification criteria are not internationally accepted, which may lead to inapplicability of their model in other studies. In addition, they did not predict the risk of future progression in patients with early CKD, focusing only on healthy subjects. More studies are needed to improve it.

## Autoimmune diseases

### Multiple sclerosis

The relationship between the eyes and the immune system is so close that there are many autoimmune diseases with ocular characteristics, such as Sjögren syndrome, inflammatory bowel disease, multiple sclerosis, and many others. The patients can present as dry eyes, uveitis, optic neuritis, etc. The studies of eyes and multiple sclerosis suggested that the thickness of the retina was thing with the development of multiple sclerosis, and measurement of retinal thickness with OCT could be a biomarker of multiple sclerosis. Based on this, among the ocular images, OCT images are currently the main image source for AI diagnosis of multiple sclerosis.

Cavaliere et al. collected OCT images from multiple sclerosis and controls. They got different regions of the retina and choroid with OCT ETDRS scan and TNSIT scan modes and calculated the variables with the highest AUC (0.97) to create a diagnosis model using a support vector machine algorithm [37]. Similarly, Martin et al. got OCT images from 48 early-stage multiple sclerosis patients and 48 healthy people [38]. They measured the thickness of each layer of retina and choroid, and tried to find regions with the greatest discriminant capacity that could be used as a classifier. The best classifier showed a great performance (sensitivity = specificity = 0.98). Their work showed that the papillomacular bundle may be the first layer affected in the early stage of multiple sclerosis, and the OCT images-based AI system could be a new direction for the early diagnosis of multiple sclerosis.

## Hematological diseases

### Anemia

Anemia is commonly defined as a low concentration of hemoglobin (Hb). Most of the anemia is easily corrected, while the key is to be detected. The “gold standard” of anemia is Hb concentration measured by venous blood samples. However, the invasive procedure is a risk of pollution for medical workers and painful for patients. Therefore, some non-invasive and painless methods of measuring Hb have been developed such as pulse oximetry [39], occlusion spectroscopy [40], photoplethysmography [41] and reflectance spectroscopy [42]. Compared to the “gold standard”, the stability and accuracy of these methods have yet to be proven.

During the physical examination, pallor of the skin mucosa such as lips and palpebral conjunctiva is often considered as an indication of anemia. Therefore, some researchers have attempted to estimate anemia status by AI through palpebral conjunctiva images. With the help of image processing programs, scientists extracted color information from segmented palpebral conjunctiva images. Early in 2007, Suner et al. took pictures of palpebral conjunctiva and got RGB values from them [43]. They developed an algorithm according to the manually delineated regions of the digital photos. This algorithm allowed for a crude calculation of the subjects’ hemoglobin concentrations. However, the quality of the images was easily influenced by the environment. Algorithms and wearable devices were developed to overcome this challenge [44, 45]. However, despite the variety of methods, detecting Hb values seemed to be difficult for AI that relied on eye images. It might be easier to diagnose anemia rather than to detect Hb values for AI. On the other hand, the association between retina and anemia was discovered [46], and researchers tried to use AI based on retinal images to diagnose anemia. Mitani et al. proposed a hypothesis that anemia could be detected from retinal images using deep learning [47]. They collected data from UK Biobank, and developed deep learning systems based on fundus images and metadata to det Hb and anemia status. Also, the combined AI system made a good performance on diabetes patients (AUC = 0.89). Along with the wide application of OCT technology in the field of ophthalmology, Wei et al. used retinal OCT images and deep learning to predict anemia for the first time [48]. Their work has led to a new level of efficacy in assessing anemia status, with a high accuracy of 98.65%.

## Others

### Sleep disorders

Obstructive sleep apnea (OSA) and narcolepsy are two main types of sleep disorders. The former is characterized by recurrent apnea during sleep, and the latter is characterized by drowsiness when awake and disturbed sleep. The diagnosis of OSA depends on a sleep breathing test, while the “gold standard” for the diagnosis of narcolepsy is the multiple sleep latency test. Relatively difficult diagnostic criteria limit the diagnosis of sleep disorders. With the deeper research of sleep disorders, the sleep status of patients with sleep disorders was gradually being understood and it had been found that electroencephalogram (EEG) and pupil size can represent such alterations [49, 50]. Based on this, Liu et al. developed a neural network method to detect OSA and narcolepsy according to EEG and pupil size [51]. The accuracy of the algorithm was above 90%.

### Hepatobiliary diseases

Previously, it appeared that ocular symptoms such as scleral yellowing due to jaundice and K-F rings due to hepatomegaly could only offer suggestive information for particular disorders. However, Xiao et al. innovated the use of ocular images in the detection of hepatobiliary diseases with AI [52]. They collected clinical data and ocular images (fundus images and slit-lamp images) of patients with seven hepatobiliary diseases from multiple centers and built an AI detection system based on the ocular images. Their algorithm has achieved good results in diseases such as cirrhosis and liver cancer (both of AUCs were over 0.83). In comparison to the AI, it was difficult for the six ophthalmologists to determine whether the subjects had hepatobiliary disease based on the ocular images, let alone diagnose the specific type of diseases. When using the heat map to interpret AI concerns, the highlighted areas are the optic disc and blood vessels of the fundus images and the conjunctiva, sclera, and iris of the slit-lamp images. This may help to discover new mechanisms of hepatobiliary diseases.

### Systemic parameters

The previous studies demonstrated that we could observe the whole body non-invasively through the eye. The above discussion shows that ocular images can be used for diabetes, cardiovascular disease, anemia, and many other systemic diseases. In 2019, Banowati et al. detected cholesterol levels by iris images with the help of AI, whose accuracy was 97.45% [53]. And in the same year, Vaghefi et al. were able to determine the smoking status of the subjects based on retinal images [54]. However, as AI is still in the developmental stage, reflecting the whole-body state through ocular images still requires careful and comprehensive research. Thus, Rim et al. did the largest study to date in a related field [55]. Collecting more than 230,000 fundus photographs, as well as setting 47 systemic parameters (age, sex, body-mass index, blood pressure, and some laboratory measurements, such as creatinine) as outputs, they evaluated the diagnostic efficacy of the AI more comprehensively. And the impact of different races on AI was also compared. Despite the poor detection efficacy of AI systems in some biomarkers, especially in external validation sets, it demonstrated the relevance of the eye to the whole body from another perspective. In addition, considering parameters with poor predictive performance, Rim et al. suggested that retinal changes may better reflect chronic diseases such as cardiovascular disease and chronic kidney disease.

## Discussion

This paper reviews the studies of ocular images-based AI in systemic diseases. Current studies are mainly focused on diseases that are very closely associated with the eye, such as diabetes, cardiovascular disease, CKD and AD. Researchers haven't often used AI to examine the connection between other diseases and the eye because there hasn't been much study on intrinsic connections. And this relies on researchers to further explore the ocular features associated with systemic diseases. Most current studies have focused on the use of ocular images to diagnose systemic diseases, with a small number being predictions of future incidence or progression, while clustering tasks are rare. This is mainly due to the level of difficulty in obtaining data. Future researchers may focus more on the task of predicting disease progression and assisting physicians in developing treatment plans.

Ocular images-based AI has benefits in several aspects, including reducing screening costs, improving efficiency and coverage, and reducing the burden on physicians. In the future, ocular images-based AI may be more widely used in screening, as it is presently used clinically to detect retinal diseases [56]. In places with limited medical resources, AI can perform routine screening by integrating software into fundus cameras. Fundus images can simultaneously detect retinal diseases, systemic diseases, and risk factors with the help of AI, which may help us to identify problems at an early or even pre-clinical stage of the disease.

In the future, many areas still require research. Firstly, apart from diagnosis, researchers can explore predicting the prognosis of diseases through eye imaging. This can help doctors in planning medical treatments. Secondly, most current researches use two-dimensional ocular images, and the acquisition of three-dimensional images from OCTA [57] or other devices [58] may be a new direction. Treating the entire eye as a single entity may help us uncover more differences in diseases. Thirdly, more innovative algorithms must be developed and incorporated to enhance disease detection performance.

Currently, the application of ocular images to detect systemic diseases confronts many challenges. First, a large number of images are needed in AI systems. Not only the construction of the model, but also testing the performance of the model requires an external validation set. In addition, the number of ocular images needed for each type of disease is currently unknown. However, for most departments, eye examinations are not routine. Therefore, to study the relationship between systemic diseases and the eyes, the acquirement of high-quality ocular images and a complete database of clinical information needs to be fully supported. This requires close collaboration between doctors in ophthalmology and other departments, as well as the assistance of AI engineers. To eliminate the interference of the number of images, the researchers must make images “better and more”.

Second, there are a lot of “black boxes” in the field of AI. There is still a great deal unknown about the mechanisms of image recognition by artificial intelligence, especially by deep learning systems. Some scientists have attempted to debunk AI systems, but no better explanatory views have emerged yet. Therefore, although some studies have attempted to represent the regions of interest of AI in the form of heat maps, we cannot understand the principles by which the system establishes the association of ocular and systemic diseases. Machine learning algorithms apart from deep learning may have better interpretations and should be applied more often to obtain more highly interpretable eye features.

Third, some rare diseases are difficult to study with AI. Based on the need for AI systems to extract features, a large number of high-quality images are indispensable to build a diagnostic system. However, this is difficult to do for some rare diseases. In these areas, it is likely that manual diagnosis will still dominate in the future. Although there are algorithms to increase the number of images by image enhancement, this is still of limited help for diversity. Multicenter studies and interpretable machine learning algorithms [59] may be the solutions.

Fourth, ophthalmic imaging devices vary widely and produce different types of images. For example, fundus images from different devices may differ in size, resolution, image format, and color. Using images from different devices to build AI models may reduce model performance. Conversely, if a model can perform well in different styles of image datasets, it indicates that the model has good robustness. Therefore, we need an algorithm or image standard to make different images easy for AI recognition.

Fifth, there are many ethical and legal issues before AI can be used in the clinical setting. There is a risk of information leaking when using AI systems because they need a lot of patient data to build them. To make sure that patient information is not utilized in other ways, strict legal restrictions and information protection measures are required. Blockchain technology has been used in data transfer to ensure that patients' personal information was not misused [60].

## Conclusion

With the progress of AI and medical big data, ocular images have already been used in the detection of endocrine, cardiovascular, neurological, renal, hematological, and many other diseases. These studies have further deepened our understanding of the eye as a reflection of the whole body. More relationships between eye and systemic diseases may be discovered and further used to enhance the effectiveness of AI. In the meantime, the intrinsic link between the eyes and many diseases is still unknown. We hope to make more progress in the AI algorithms and physiological and pathological mechanisms to reveal the true connection between ocular and systemic diseases.

### Supplementary Information

**Additional file 1.** **Table S1** Summary of the studies about ocular images-based AI for systemic diseases and parameters.

## Data Availability

Not applicable.

## Abbreviations

AI: Artificial intelligence
CNN: Convolutional neural network
OCT: Optical coherence tomography
OCT-A: OCT-angiography
AUC: Area under the receiver operating characteristic curve
RGCs: Retinal ganglion cells
RNFL: Retinal nerve fibers layer
AD: Alzheimer’s disease
CKD: Chronic kidney disease
TINUS: Tubulointerstitial nephritis uveitis syndrome
AMD: Age-related macular degeneration
Hb: Hemoglobin
OSA: Obstructive sleep apnea
EEG: Electroencephalogram
